# A Computational Model of Working Memory Based on Spike-Timing-Dependent Plasticity

**DOI:** 10.3389/fncom.2021.630999

**Published:** 2021-04-21

**Authors:** Qiu-Sheng Huang, Hui Wei

**Affiliations:** Laboratory of Cognitive Model and Algorithm, Shanghai Key Laboratory of Data Science, Department of Computer Science, Fudan University, Shanghai, China

**Keywords:** spike-timing-dependent plasticity, working memory, computational model, synaptic plasticity, spiking neural network

## Abstract

Working memory is closely involved in various cognitive activities, but its neural mechanism is still under exploration. The mainstream view has long been that persistent activity is the neural basis of working memory, but recent experiments have observed that activity-silent memory can also be correctly recalled. The underlying mechanism of activity-silent memory is considered to be an alternative scheme that rejects the theory of persistent activity. We propose a working memory model based on spike-timing-dependent plasticity (STDP). Different from models based on spike-rate coding, our model adopts temporal patterns of action potentials to represent information, so it can flexibly encode new memory representation. The model can work in both persistent and silent states, i.e., it is compatible with both of these seemingly conflicting neural mechanisms. We conducted a simulation experiment, and the results are similar to the real experimental results, which suggests that our model is plausible in biology.

## Introduction

Working memory is the ability to preserve information for further processing (Baddeley, 2010). It participates in complex tasks such as reasoning, understanding, and learning, and is an important part of the cognitive function. Reflecting its importance, working memory has been a research object for many years, but its neural mechanism is still not fully understood. As early as 1971, a study on short-term memory by Fuster and Alexander reported that some neurons of the prefrontal cortex maintained a higher firing rate in the delay period than in the intertrial period (Fuster and Alexander, 1971). Similar phenomena were observed in subsequent experiments (Funahashi et al., 1989; Nakamura and Kubota, 1995; Watanabe and Funahashi, 2007; Wimmer et al., 2014). Since there is no external cue stimulus during the delay period, this spontaneous activity is likely related to the memory content. Some studies have found that if researchers try to inhibit the persistent activity of neurons during the delay period, the accuracy of task recall will decrease (Quintana et al., 1989). More importantly, a machine learning model trained by the neuronal spike data collected during persistent activity can predict the behavior of animals in the recall phase (Masse et al., 2017). Therefore, persistent activity is considered the most reasonable candidate mechanism for working memory, and some computational models based on the interconnection of neurons have been proposed. For example, in the ring model (Compte et al., 2000), the connection strength of neurons is determined according to their stimulus selectivity, and the input information is maintained by mutual excitation of adjacent neurons. Similar models reproduce the phenomena observed in neurobiology experiments, but they are usually only suitable for spatial orientation memory tasks.

Recent studies have found that working memory can be independent of persistent activities, which provides a new way to understand its neural mechanism. For example, under dual tasks, even when an experimental subject makes the correct choice, the spatial selectivity of delayed activities is significantly reduced (Watanabe and Funahashi, 2014), indicating that working memory can be retained in the “activity silence” neural state (Stokes, 2015). Another study found that transient gamma oscillations are accompanied by reactivation of coding and sensory information (Lundqvist et al., 2016), which means gamma oscillations may also be the basis of working memory. Accordingly, models independent of persistent activities have been proposed, in which information is stored by synaptic changes rather than neural activity. This change occurs through specific synaptic mechanisms, such as presynaptic calcium residue (Mongillo et al., 2008) and short-term enhancement (Fiebig and Lansner, 2017; Mi et al., 2017).

Since the observed experimental phenomena are quite different, these two mechanisms are usually considered incompatible. However, because of differences in species types, individual conditions, and experimental methods, the observed phenomena may not address the same problem. Even in research related to persistent activity, only a part of the neurons related to the experimental task show persistent activity, and they may exhibit complex, diverse temporal patterns (Watanabe and Funahashi, 2007). This suggests that these two mechanisms may coexist in the brain or stem from a deeper mechanism. Researchers have recently attempted to integrate the two mechanisms into a unified framework (Kaminski and Rutishauser, 2020). Such work provides a new perspective on the neural mechanism of working memory, and its significance is obvious.

A variety of synaptic plasticity mechanisms have been applied to working memory models. Past models mostly adopted the plasticity mechanism based on facilitation. Since synaptic reinforcement is not selective, these models cannot explain the encoding of novel association (Fiebig and Lansner, 2017). To overcome these shortcomings, some researchers have adopted Hebbian’s rule (Fiebig and Lansner, 2017), whose idea is to use a selective synaptic enhancement mechanism. Following this idea, we propose a working memory model based on STDP. Under the influence of STDP, the neurons can enhance the causal connections and ignore the irrelevant connections (Markram et al., 2012), thus supporting more flexible encoding schemes. Although the STDP rule is usually applied to a computational model on a longer time scale, some experimental phenomena under this rule at different time scales have been observed (Caporale and Dan, 2008). Therefore, it is reasonable to apply STDP to a computational model on a smaller time scale. We conducted three experiments to evaluate our model, following the common delay-match-to-sample (DMS) paradigm. The first two experiments evaluated the model under persistent and silent activity, and the last was compared to a psychology experiment. The experimental results show that memory items in our model can be well recalled in both neural activity states. The simulation results are similar to real experiment results, suggesting that our model is reasonable and plausible in biology.

## Materials and Methods

### Neural Circuit Model

**FIGURE 1:**
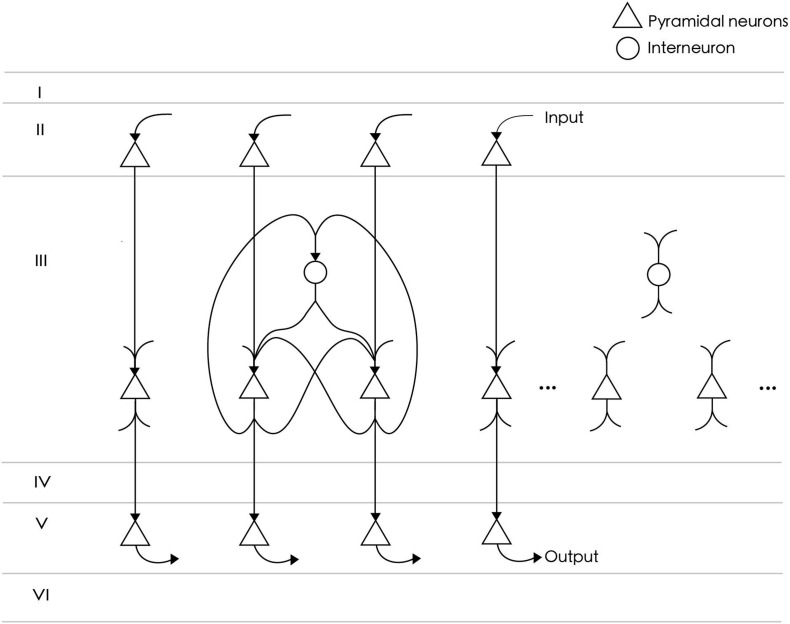
The neural circuit consists of 24 excitatory neurons (triangles) and six inhibitory neurons (circles). Not all neurons are shown in the figure. Among them, 15 excitatory neurons directly receive spike signals from upstream input neurons and are connected to downstream readout neurons. All excitatory neurons are fully connected and project to inhibitory neurons with an 80% probability. There is no connection between inhibitory neurons.

The neural circuit model represents a functional cluster of neurons in the prefrontal cortex for single-item memory (Figure 1). The circuit consists of *N*_*E*_ = 24 excitatory neurons and *N*_*I*_ = 6 inhibitory neurons. The number of excitatory neurons is four times that of inhibitory neurons, which meets the proportion of neurobiological findings (Markram et al., 2004). All excitatory neurons are fully connected, and each connection consists of *S*_*EE*_ = 4 synapses. The strengths of these synapses are initially random values between 0 and 0.14. The connection probability between excitatory neurons and inhibitory neurons is 0.8, and their connection strength is fixed. Inhibitory neurons are used to inhibit excitatory neurons to prevent overexcitation, so there is no connection between them. According to the reports of axon conduction delays in cat and rabbit cerebral cortex (Miller, 1975; Swadlow, 1990), the average conduction delay of connections in our model is set at about 12 ms. Specifically, the conduction delays of connections between excitatory neurons are set to $d_{i\,j}^{k}=R_{E\,E}+k*3$, where *R*_*EE*_ is a random value between 3 and 12, and *k* = 0, 1, 2, 3 is the index corresponding to four synapses of each connection. The delays between inhibitory neurons are random values between 1 and 20 ms. Among the 24 excitatory neurons in the network model, *N*_*Input*_ = 15 are used to receive the input of a spike sequence, and as output neurons to extract memory items.

### Neuron Model

We used the Izhikevich neuron model to simulate the firing behavior of real neurons (Izhikevich, 2003). It combines the advantages of the leaky integrate-and-fire and Hodgkin-Huxley models and can simulate various spiking patterns with relatively simple calculation. The model is represented as follows:

(1) $$v^{\prime }=0.04\,v^{2}+5\,v+140-u+I,$$

(2) $$u^{\prime }=a\,\left(b\,v-u\right),$$

(3) $$\mathrm{if}\,v>30\,\text{mV},\mathrm{then}\,\left\{ \begin{array}{c} v\leftarrow c\\ u\leftarrow u+d \end{array}\right.$$

where *v* is the membrane potential of the neuron, *u* is the recovery variable of the membrane potential. *I* is the current input to the neuron, which includes the current from other neurons in the model and noise current, that is:

(4) $$I=I_{\text{input}}+I_{\text{noise}},$$

where *I*_noise_ is Gaussian noise with mean ranging from 1.5 to 2 and variance 1.8. It can be reasonably assumed as irrelevant input from nearby cortical tissues. *a*,*b*,*c*,*d* in Equation 2 and 3 are dimensionless parameters. The model can reproduce different spiking patterns according to the parameter settings. The parameters of excitatory neurons in our model are *a* = 0.02,*b* = 0.2,*c* = −65,*d* = 8, and those of inhibitory neurons are *a* = 0.04,*b* = 0.24,*c* = −65,*d* = 2.

### Synapse Model

**FIGURE 2:**
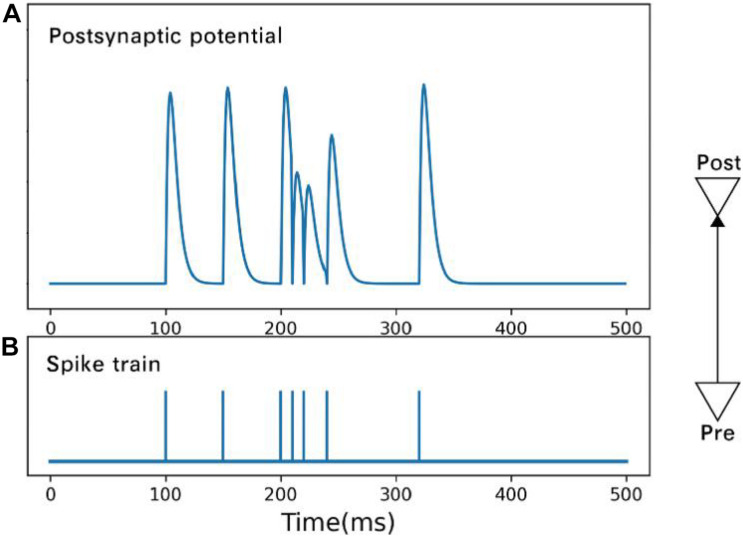
Postsynaptic potential **(A)** is changed by spikes **(B)** of different frequencies. Under the condition of high-frequency spikes, the rising amplitude of postsynaptic membrane potential caused by a subsequent spike drops.

Synapses are the key parts of neurons, which pass spiking signals to each other. In the main kind of synapse in the nervous system of higher vertebrates, the chemical synapse, an action potential makes the terminal of the presynaptic axon release neurotransmitters, which diffuse across the synaptic cleft and bind to receptor proteins on the postsynaptic membrane, causing the increase or decrease of postsynaptic membrane potential (Kandel et al., 2012). Following the design of Bohte et al. (2002), we describe the time-dependent change of postsynaptic potential caused by an action potential as:

(5) $$V_{P\,S\,P}\,\left(t\right)=\frac{t}{\tau }\,e^{1-t/\tau },$$

where τ is a time constant, which we set to 4. The equation describes the potential changes caused by a single spike, but in real situations, the high-frequency spikes on a single synapse may cause multiple potential changes to overlap. When synapses fire multiple spikes in a short period, the amplitude of the changes of the postsynaptic membrane potential caused by each spike will steadily decrease due to the depletion of neurotransmitters, which gradually return to normal over time. This effectively reduces the possibility of over-firing of the whole neural circuit. We refer to the model of Mongillo et al. (2008) to describe this process:

(6) $$x^{\prime }=\frac{1-x}{\tau_{D}}-u\,x\,\delta \,\left(t-t_{s\,p}\right),$$

(7) $$u^{\prime }=\frac{U-u}{\tau_{F}}+U\,\left(1-u\right)\,\delta \,\left(t-t_{s\,p}\right),$$

where *x* is the available resources of the synapse; *u* is the utilization rate of resources by each spike;=′d/dt; δ is the Dirac function; *t*,*t*_*sp*_ are the current time and firing time, respectively, of a spike; *U* is the benchmark level of utilization; and τ_*F*_,τ_*D*_ are recovery time parameters set to 20 and 50, respectively, in our model. The transmission efficiency of a synapse is expressed as *xu*. Therefore, the final postsynaptic membrane potential at time *t* can be expressed as:

(8) $$V_{P\,S\,P}\,\left(t\right)=\sum_{i=1}^{n}u_{i}\,x_{i}\,\frac{t-t_{i}}{\tau }\,e^{1-\left(t-t_{i}\right)/\tau },$$

where *u*_*i*_,*x*_*i*_,*t*_*i*_ are the available resources, utilization rate, and arrival time, respectively, of the *ith* spike. Figure 2 shows the postsynaptic potential produced by spikes of different frequencies.

### Spike-Timing-Dependent Plasticity

Synaptic strength may change under certain induction events (Gustafsson et al., 1989). Research in the late 1990s shows that the difference of firing time between pre- and post-synaptic neurons has a significant impact on the change of synaptic strength (Gerstner et al., 1996; Markram et al., 1997; Bi and Poo, 1998). The synaptic strength is enhanced when the pre-synaptic neuron fires slightly before the post-synaptic neuron and depressed when they fire in reverse order. Such synaptic plasticity is called spike-timing-dependent plasticity (STDP). The relationship between the difference of firing time and the change of synaptic strength is expressed by STDP function, which can be obtained from spiking activities with appropriate modeling (Song et al., 2015; Robinson et al., 2016). STDP is different at different synapses. It exists not only in excitatory synapses but also in inhibitory synapses (Haas et al., 2006). Hence, there are many forms of STDP function. We apply a traditional form to the model (Song et al., 2000; Bi and Poo, 2001). The STDP function is:

(9) $$\Delta \,W\,\left(\Delta \,t\right)=\left\{ \begin{array}{c} A_{+}\,e\,x\,p\,\left(\Delta \,t/\tau_{+}\right)\,i\,f\,\Delta \,t\,<0\\ -A_{-}\,e\,x\,p\,\left(\Delta \,t/\tau_{-}\right)\,i\,f\,\Delta \,t\,\geq 0 \end{array}\right.,$$

where Δ*t* is the time difference between the pre- and post-synaptic neuron; τ_+_, τ_−_ are parameters that affect the time windows; and *A*_+_,*A*_−_ are the maximum degree of enhancement or weakness, respectively. The spike pairing scheme in this model is the nearest-neighbor scheme (Morrison et al., 2007), i.e., a presynaptic spike is paired with only the last postsynaptic spike, and a postsynaptic spike is paired with only the last presynaptic spike.

Studies have shown that STDP can cause neurons to synchronize or desynchronize (Talathi et al., 2008; Popovych and Tass, 2012; Popovych et al., 2013; Borges et al., 2016, 2017; Lameu et al., 2018), and the synchronization of neural activity is bound up with brain information processing (Baravalle et al., 2019). According to the encoding scheme in this model, the best decoding case is that the neurons are synchronized with specific phases (see section “Encoding and Decoding”). However, strict synchronization, that is, all neurons fire at the same time, is not ideal, because the order information of the spikes is missed. Previous researches usually set the potentiation and depression windows in STDP to be approximately the same size. After testing, we found that this can easily lead to strict synchronization. Therefore, we increased the depression window. The STDP parameters in our model are:*A*_+_ = 1, *A*_−_ = −1, τ_+_ = 3, τ_−_ = 18.

The synaptic strength is updated according to *W*_*t*_ = *W*_*t*−1_ + ηΔ*W*, where η is the learning rate. The learning rate should be higher in the working memory model because the sample needs to be memorized quickly. On the other hand, the excessive value will lead to unstable learning. After testing, we set the learning rate to 0.2.

### Encoding and Decoding

How the brain encodes information is a key point for the working memory model. We focus on the temporal information carried by the spike, just like our previous work (Wei and Du, 2019). The encoding scheme of memory items in this model comes from rank order coding. This strategy is based on population coding, in which the information is represented by a group of neurons. The idea of order coding is that the firing time order of neurons is critical, and not the exact firing latency time (Gautrais and Thorpe, 1998). This simple idea is plausible in biology. The time from receiving an input to excitation is related to the input’s intensity (Heil, 2004; Gollisch and Meister, 2008). Therefore, when there is a stimulus input, neurons encoding different dimensions produce spikes with various excitation latencies to generate a specific spike train.

Applying sequential coding to a computational model has at least two advantages. First, it helps to quickly and accurately identify spike trains. In theory, the complete recognition time of order coding is merely the arrival time difference between the first and last spike, while traditional rate-based coding must count the number of spikes over a time. To reduce the statistical error, the time window is often large; hence, it takes more time to discriminate different spike trains. Second, order coding has high efficiency, and it can represent much information with only a few neurons. If each neuron only fires one spike, then *n* neurons with order coding can represent *n*! states, while rate-based coding scheme can represent at most *n+1* states (Gautrais and Thorpe, 1998).

Although the specific firing time of each spike is immaterial to the order coding scheme, the minimum distinguishable time between two spikes must be considered in practice. If the firing times of two spikes are close, then their order may be wrongly resolved under the influence of errors. To simplify the model and improve the recognition accuracy, we add a limitation based on order coding: only one neuron fires a spike during any 4 ms. In this way, the firing time of each neuron can be calculated from a spike train. Considering the case of three neurons, the firing times of neurons 0–2, corresponding to the sequence (1, 3, 2), are 4 ms, 12 ms, and 8 ms.

**FIGURE 3:**
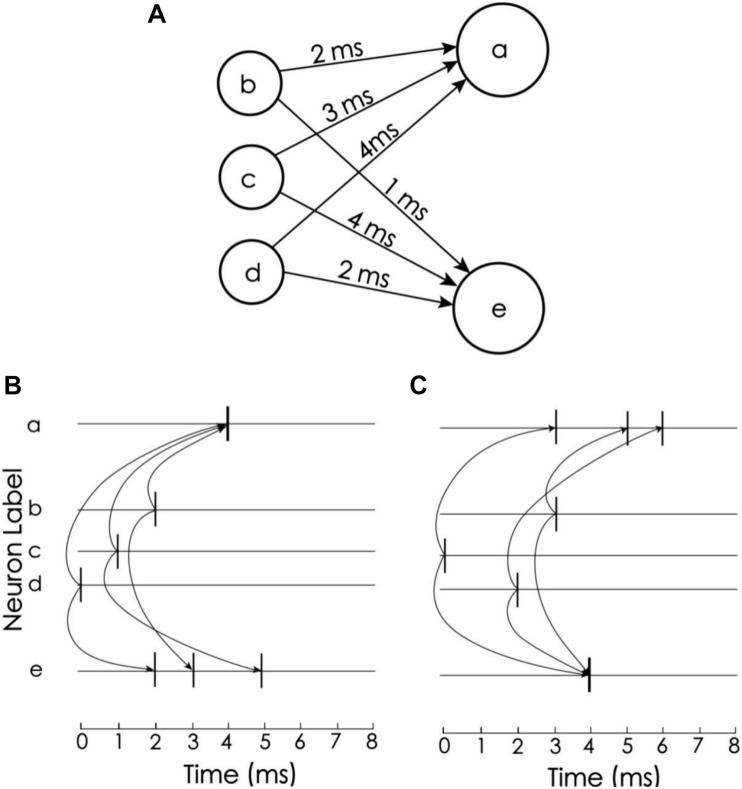
Spike train pattern detection. **(A)** Neurons a and e receive spike inputs from neurons b, c, and d, but the synaptic transport delays of the connections are different. **(B)** The transport delay of the synapse of neuron a causes the spikes of the three upstream neurons to arrive at the same time. Therefore, neuron a is activated and e is not. **(C)** Neuron e is excited but a is not (Adapted from Izhikevich, 2006).

In this coding scheme, the information can be easily recognized by the corresponding detection neurons. The idea of decoding comes from Izhikevich’s polychronization concept (Izhikevich, 2006), i.e., to set the propagation delay so that a train of spikes with a given order arrives at the corresponding detection neurons at the same time. In the proposed model, there are 15 neurons to encode and decode memory items, but for simplicity, we consider only three neurons to explain the idea (Figure 3). Neurons a and e are detection neurons of different spike trains, and the respective propagation delays between a and e and neurons b, c, and d are 2 ms, 3 ms, 4 ms and 1 ms, 4 ms, 2 ms (Figure 3A). When the firing times of b, c, and d are 2 ms, 1 ms, and 0 ms, respectively (Figure 3B), these spikes arrive at neuron a at the same time, causing it to excite. For neuron e, these spikes arrive at different times. Due to the leakage of the membrane current, these spikes cannot accumulate enough current to excite neuron e. Conversely, if the firing times of b, c, and d are 3 ms, 0 ms, and 2 ms, then e is excited and a is not. Therefore, neurons a and e can be used to detect spike train patterns (2, 1, 0) and (3, 0, 2), respectively.

### Experimental Procedure

**FIGURE 4:**
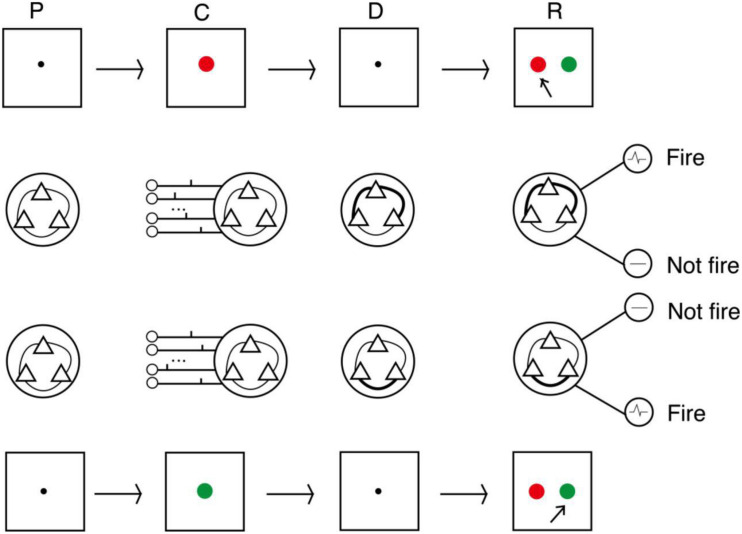
Process of DMS experiment. In the cue period, red and green color samples were encoded by 15 neurons into two input patterns of spike trains. In the delay period, the strengths of synaptic connections were adjusted by the STDP rule. In the response period, samples were read out by corresponding detecting neurons (P, preparation period; C, cue period; D, delay period; R, response period).

We conducted three experiments to evaluate the proposed model. The procedures followed the DMS task paradigm, and the sample stimuli were two colors (red and green). The procedure is shown in Figure 4. The first second of an experiment was a preparation period, when the subjects prepared to receive the sample stimulus prompt. At this time, there was no external input, and the model was only affected by random noise current from other neurons in the cerebral cortex. The next second was the cue period. Subjects were prompted by either red or green, and the spike train that encoded the color was loaded into the model. The sample stimulus disappeared at the beginning of the delay period. After a 3-s delay, subjects were asked to recall the sample. This was done by imposing a non-specific recall signal to the model, e.g., an extra noise current. The input sample stimuli were encoded by 15 neurons as two different spike train patterns, and decoding was performed by detection neurons corresponding to the two patterns. In the response period, the activities of detection neurons within 0.5 s were counted, and the sample corresponding to the more active neuron was the recalled sample.

## Results

### Memory Under Persistent Activity

In electrophysiological experiments of working memory, it is usually observed that some neurons relevant to the experiment maintain a firing rate significantly higher than the reference level during the delay period, and this is known as persistent activity. Because of its strong correlation with the memory task, persistent activity theory is dominant in explaining the neural mechanism of working memory. The first experiment tested whether the model can maintain memory and display persistent activity during the delay period. For this purpose, we increased the mean of the noise current by 1.5 for each excitatory neuron. This leads to a slight depolarization, which makes the neuron likely to fire.

**FIGURE 5:**
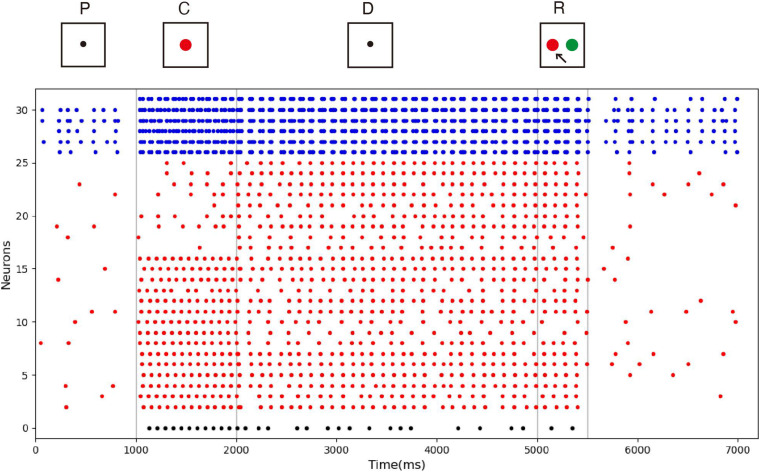
Spike raster of neural circuit under persistent activity. Black dots, red dots, and blue dots represent detection neurons, excitatory neurons, and inhibitory neurons, respectively. The meanings of P, C, D, and R are consistent with those in Figure 4. The red sample is loaded into the model during the cue period. In the delayed period, there is no sample stimulus, but neuron 0 is still activated until the response period, indicating that sample can still be recalled. Neuron no. 1, which detects green, remains silent during the experiment.

The spike raster of the whole neuronal population during the experiment is shown in Figure 5. Neurons 0 and 1 (black) are the detection neurons; neurons 2–25 (red) are excitatory neurons, including 15 neurons (2–16) that receive input; and neurons 26–31 (blue) are inhibitory neurons. The first second is the initial ground state of the model. During this period, the neurons are driven by the noise current from the surrounding cortex, so they irregularly fire at low frequency. During the cue period of the first second to the second second, a sample stimulus (red) is loaded into the network model, and the network continuously receives the spike train encoded by 15 input neurons. No. 0 neuron is activated at the same time as the detection neuron of red color, while no. 1 neuron, which detects the green color stimulus, is completely silent. The delay period is from the second to fifth second, during which the excitatory neurons show sustained excitation. Although the input of the sample stimulus has disappeared, neuron no. 0 is still activated frequently, which indicates that memory items still exist in the model network. After the response period, the activity of the whole group of neurons decreases and returns to the ground state of low frequency and irregular excitation.

Although there is no unified definition of persistent activity, it is generally believed that persistent activity is related to memory information rather than meaningless random activity. Therefore, some researchers use machine learning methods such as support vector machine (SVM) to analyze the activities of neurons related to working memory. Here, we trained a simple SVM to decode the activity data in the delay period of the model. The firing rates of each neuron were the average spiking activity in 500-ms bins sampled at 200-ms sliding intervals. These data were used as samples to train the SVM.

**FIGURE 6:**
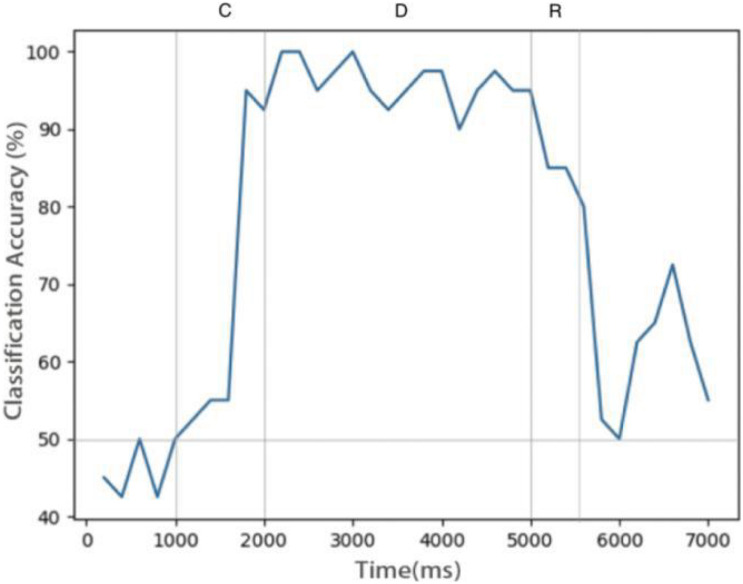
Decoding result of SVM classifier trained by data of neurons during delay. Decoding accuracy expected by chance is 50%.

Figure 6 shows the relationship between decoding accuracy and time. Since there are only two types of stimulus, the probability of decoding by chance is 50%. It can be seen that after the end of the sample cue period, the decoding accuracy rate reaches more than 90% and remains there for the whole delay period, which proves that the persistent activity in the model is indeed related to the stimulus information, rather than purely random activity. During the delay period, the accuracy rate decreases gradually, indicating that the memory is gradually blurred. This is done entirely through STDP, with no other mechanism. We also see that SVM can decode correctly from the release activity after the end of the response period, which indicates that there is still some residual memory.

### Memory Under “Silent” State

While the mainstream view is that persistent activity underlines working memory, some believe that memory information can be stored in a “silent” neuron state. Therefore, our second experiment tested whether the model can maintain memory in the silent state. This experiment was much like the first, but the mean of the noise current of each excitatory neuron was reduced by 0.3, so they could not form obvious persistent activities. To show another sample stimulus, green was used as an input. Note that the choice of sample stimulus does not affect the model’s performance.

**FIGURE 7:**
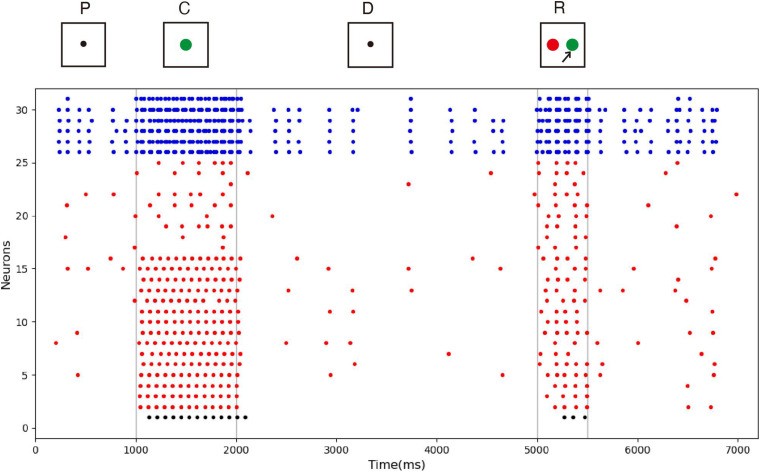
Spike raster of neural circuit under “silent” state. The meanings of the color of these dots are the same as those in Figure 5. In this experiment, the sample stimulus is green, which is detected by neuron no. 1. The excitatory neurons are inhibited in the delayed period, and they fire at a quite low rate. However, neuron no. 1 is activated by the recall signal in the response period, indicating that sample can still be recalled.

**FIGURE 8:**
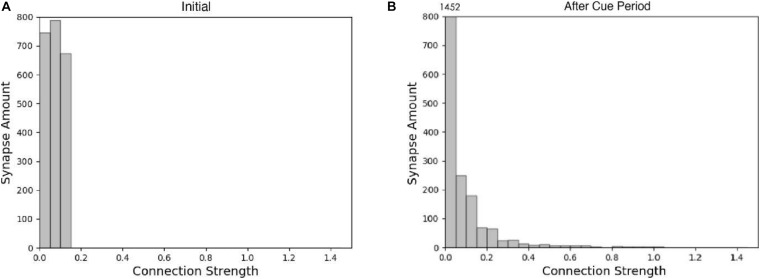
Histogram of synaptic connection strength. **(A)** Initial; **(B)** after sample presentation.

The spike raster of the whole neuron group in the simulation experiment is shown in Figure 7. The first 2 s of the experiment are similar to experiment 1. After the cue period, the neuronal population does not show sustained excitation as in experiment 1, but returns to the low-frequency ground state due to the inhibition. However, despite the lack of sustained activity during the delay period, neuron no. 1, which detects green, is activated under the stimulation of a non-specific recall signal during the response period, implying that the sample can still be accurately recalled. This indicates that our model preserves information through changes in synaptic connections rather than persistent activity. During the cue period, the information of relative firing time difference between neurons is preserved in synaptic connections by STDP. The recall signal in the response period reproduces the spike pattern of the sample stimulus, which can be discriminated by the detection neurons. Figure 8 shows the changes in the strengths of synaptic connections after the sample loading. In the initial state, the synaptic connection strength is set at about 0.07 on average. After learning, most of the synaptic strength is close to zero, and only a small part is greater than 0.2, showing a lognormal distribution.

### Simulation Experiment

In Experiment 3, we applied the model to continuous DMS tasks and compared the data to that of a pigeon DMS experiment by Roberts and Kraemer (1982). In their experiment, pigeons were kept in cages with three buttons, placed side by side. After the experiment started, the middle button lit and displayed green or red as the sample stimulus. Next were delay and response periods. In the response period, the left and right buttons were illuminated, respectively, in green and red as alternative samples. A pigeon that made the right choice was rewarded, and the experiment was finished; otherwise, the experiment finished directly. The next experiment started after a short interval. Roberts and Kraemer explored the accuracy of pigeon matching with different combinations of delay intervals and experiment intervals. Our simulation experiment is basically consistent with their experiment, except the sample prompt period and experimental interval were reduced to one-eighth of the original to meet the time scale of our model.

**FIGURE 9:**
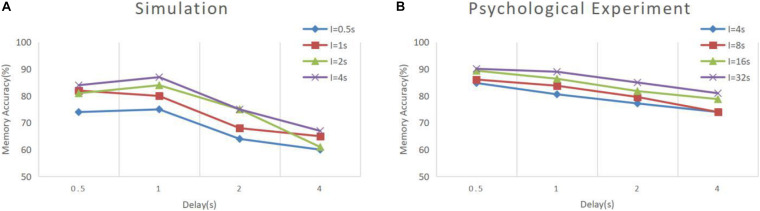
Memory performance comparison between simulation and psychological experiment (Roberts and Kraemer, 1982). **(A)** Simulation result of our model, where “I” denotes the intertrial interval. **(B)** Result of psychological experiment. The simulation was conducted under the persistent activity state, and the noise current setting was consistent with experiment 1. All the combinations of experimental conditions were repeated 100 times to calculate the accuracy (**B** is drawn according to the literature).

**FIGURE 10:**
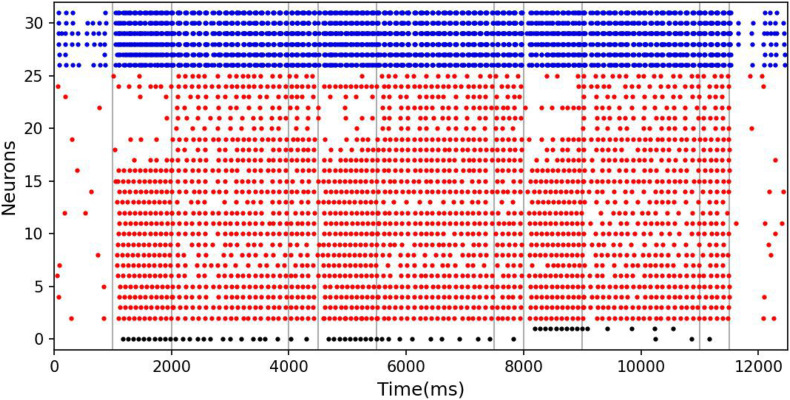
The interference between different memories. The meanings of the color of these dots are the same as those in Figure 5. In the third task, although the memory is correct at the beginning of the delay period, the response period recalls the previous sample rather than the current sample.

The results of model simulation are shown in Figure 9A. For comparison, we draw Figure 9B based on the experimental data of Roberts and Kraemer. It can be seen that the simulation results are consistent with the trend reflected by the real experiment. Each line in Figure 9 shows a downward trend, and the line with the longer experimental interval is placed at the upper position. This indicates that the recall accuracy decreases with the increase of the delay period, and increases with the intertrial interval. It is not surprising that the accuracy drops over time. Under the interference of noise, the synaptic connections formed in the cue period gradually becomes disordered, and finally, the sample cannot be recalled. The increase of accuracy with the intertrial interval may be related to the interference between memories. To test this, we conducted another experiment. We used our model to perform the DMS task three times in succession. The first two samples were the same but different from the third. The experiment was under the persistent activity state, and the delay interval and the intertrial interval were 2000 ms, 0 ms, respectively. We find that there is a possibility that the first two samples interfere with the third sample, as shown in Figure 10.

It can be seen that our model has greater volatility and does not strictly follow the trend under some combinations of conditions. We deduce that this is due to the limitations of the small scale of the model. We simulate a memory circuit consisting of only 30 neurons, which is vulnerable to noise currents. The number of neurons in the brain is much greater, and there are many redundant functional circuits, so the psychological experimental performance is more stable. We believe that this difference will be greatly reduced if multiple circuits are combined in a larger memory network.

## Conclusion and Discussion

We proposed a working memory neural circuit model based on STDP, an extension of Hebbian’s rule, which is considered an important learning rule in the brain’s nervous system. It was verified in electrophysiological experiments on multiple time scales. Our experiment explored the feasibility of STDP as a mechanism of working memory and found that it could form a specific synaptic connection state to maintain memory items in a short time scale. The connection state could reproduce the spike pattern of the memory item with non-specific recall signals. In addition, due to the coding scheme based on the spike time information, the model can memorize items flexibly and efficiently without prior training. We tested the model in states of persistent activity and silence, and both could correctly recall the memory item in the response period. We compared the simulation data to real experimental data, and the results were similar, indicating that our model is biologically reasonable.

The internal mechanism of working memory is still under exploration. The traditional view is that persistent activity is the basis of working memory, but experimental phenomena observed in recent years suggest the possibility that working memory can be preserved in the connection state between neurons without the continuous firing of neurons. Persistent activity theory and connectivity theory are usually regarded as opposing views, but some researchers have integrated them, and think that both persistent and non-persistent mechanisms may exist in the brain. Our model supports this view. However, as pointed out before, memory stored in the silent state is more energy-saving, more stable, and less susceptible to interference than in the persistent activity state (Lundqvist et al., 2018). So, why does the brain not preserve all memory in a silent state? A reasonable explanation is that memory can be read more quickly in a state of persistent activity. In our model, memory reading in the silent state takes time, because the signal must pass to the circuit, and memory information is then passed to the downstream neurons. In a state of persistent activity, the memory content is ready to be read, so there is no delay. In some psychology theories, working memory and attention are considered to be closely related, and memory can be divided into different states according to the attention distribution (Cowan, 1988; Engle et al., 1999; Oberauer, 2002). By distinguishing different states, the brain manages working memory more efficiently. In our model, working memory is preserved through the synaptic connection state, and persistent activity may be an accessory produced by the brain for faster reading during the memory process, suggesting that the phenomena of persistent activity and the silent state may differ according to conditions in the same neural mechanism. Our study provides conjecture for integrating the two mechanisms.

## Data Availability Statement

The original contributions presented in the study are included in the article/supplementary material, further inquiries can be directed to the corresponding author.

## Author Contributions

HW carried out the computational neuroscience model and neural circuit design studies, experiment design, and writing the manuscript. Q-SH designed algorithms, coded simulation program and collected the experimental data, and wrote the manuscript. All authors read and approved the final manuscript.

## Conflict of Interest

The authors declare that the research was conducted in the absence of any commercial or financial relationships that could be construed as a potential conflict of interest.
